# Higher levels of self-efficacy and readiness for a future career among Spanish-speaking physician assistant students after their volunteer work at a student-run free clinic in the United States

**DOI:** 10.3352/jeehp.2019.16.27

**Published:** 2019-09-06

**Authors:** Shannon Weaver, Zainub Hussaini, Virginia Lynn Valentin, Samin Panahi, Sarah Elizabeth Levitt, Jeanie Ashby, Akiko Kamimura

**Affiliations:** 1 Division of Physician Assistant Studies, University of Utah, Salt Lake City, UT, USA; 2 Department of Sociology, University of Utah, Salt Lake City, UT, USA; 3 Department of Nutrition and Integrative Physiology, University of Utah, Salt Lake City, UT, USA; 4 Maliheh Free Clinic, Salt Lake City, UT, USA; Hallym University, Korea

**Keywords:** Physician assistants, Medically uninsured, Primary health care, Volunteers, United States

## Abstract

Volunteering at a free clinic may influence career choice among health profession students. The purpose of this study was to explore knowledge, skills, attitudes, self-efficacy, interest in future work with the underserved, and interest in primary care among physician assistant (PA) students through an analysis of demographic characteristics of PA students at a student-run free clinic in the United States. Data were collected from 56 PA students through a quantitative survey in October 2018 after their participation at a student-run free clinic in Salt Lake City, Utah, in the intermountain west region of the USA. Out of the 3 sub-scales (attitudes, effect, and readiness), students responded most positively to items exploring the effect of their experiences of volunteering at the free clinic. Students who spoke Spanish showed higher levels of self-efficacy and readiness for a future career than non-Spanish speakers.

Physician assistants (PAs) are medical providers with a master’s degree who examine, diagnose, and treat patients under the supervision of medical doctors in the United States. A bachelor’s degree and clinical experience are required to be eligible to enter PA school, which then constitutes 2 years of training, including a didactic year and a clinical year [1]. While there is a perception that the majority of PAs work in primary care, there has been a trend toward specialization, with only 26% of PAs nationwide working in primary care [2]. Although previous studies have examined medical students’ volunteer experiences at free clinics, to the best of our knowledge, only 2 studies have evaluated PA students’ experiences at free clinics. One study revealed that PA students who volunteered at a homeless-shelter free clinic were more likely to be interested in primary care after graduation [3], while another study showed that volunteering as lifestyle counselors during PA school served as a beneficial clinical opportunity [4].

The purpose of this study was to explore knowledge, skills, attitudes, self-efficacy, interest in future work with the underserved, and interest in primary care among PA students who volunteered at a student-run free clinic in the United States.

## Ethics statement

The Institutional Review Board of the University of Utah approved the study protocol (IRB approval no., 00072275). Informed consent was obtained from the participating students.

## Study design

This was an observational study based on a questionnaire survey after subjects had experienced volunteering at a free clinic that serves patients with incomes up to 150% of the federal poverty line.

## Study participants and data collection

This study was conducted at a PA program at the University of Utah, Salt Lake City, Utah, in the intermountain west region of the USA. Students at this program can volunteer at a PA student-run free clinic on Thursday nights. Data were collected from 56 PA students at the University of Utah using a quantitative approach (a survey) in October 2018. The survey response rate was 45% for first-year students (n=18) and 60% for second-year students (n=38) (Supplement 1).

## Questionnaire survey

Students’ self-perceptions of knowledge, skills, and attitudes toward working with the underserved, perceived self-efficacy of working with underserved patients, and interest in primary care were measured using a scale developed by Smith et al. [5]. The original scale focused on homeless populations. For this study, the word “homeless” was replaced by “the underserved.” The scale has 15 items with 6 sub-scales asking participants to rate the effect of volunteering at the free clinic: knowledge (4 items, Cronbach α=0.891; e.g., “My degree of knowledge about the problems of the underserved”); skills (2 items, Cronbach α=0.873; e.g., “My clinical skills in the care of the underserved”); attitudes (2 items, Cronbach α=0.945); self-efficacy (5-items, Cronbach α=0.923; e.g., “I feel capable of caring for the underserved”); interest in future work with the underserved (1 item, “My interest in working with the underserved after I graduate”); and interest in primary care (1 item, “My interest in being a primary care physician assistant”). A 7-point Likert scale (1=not at all, 7=a great deal) was used. Scoring was based on the mean of the items in the same sub-scale. The survey questionnaire is presented in Appendix 1.

## Data analysis

The response data were analyzed using IBM SPSS ver. 25.0 (IBM Corp., Armonk, NY, USA). Descriptive statistics included frequency and percentage for categorical variables and mean and standard deviation for continuous variables. General linear model regression analysis was performed to examine the associations between the impact of volunteering at a free clinic and demographic characteristics. Collinearity was evaluated using the variance inflation factor, and no significant collinearity was found.

Table 1 presents the sociodemographic characteristics of 56 first- and second-year student participants out of 87 subjects (64.4%). Thirty-two participants were female (57.1%). Nineteen participants were aged under 30 (33.9%). Twenty-two participants had 5 years or less of clinical experience (39.3%). Twenty-five participants could communicate with patients in Spanish (44.6%). Table 2 presents descriptive statistics. Out of the 3 sub-scales (attitudes, effect, and readiness), students responded most positively to items exploring the effect of their experiences of volunteering at the free clinic. Multiple linear regression analysis was conducted to examine the association between sociodemographic characteristics (independent variables) and attitude, effect, and readiness (dependent variables) (Table 3). Being able to speak Spanish was the only influencing factor; speaking Spanish was associated with higher levels of self-efficacy (P<0.01), effect (P<0.01), and readiness (P<0.05) in regard to volunteering at the free clinic.

The major finding of the quantitative analysis was that speaking Spanish was associated with higher levels of self-efficacy and readiness to be a PA. While there is a lack of information surrounding the success of bilingual health profession students, significant data exist regarding the impact of language barriers on patient-provider relations. A study found that language barriers in a clinical setting could cause many obstacles to providing the best care [6].

In conclusion, volunteering at a free clinic during PA school was correlated with increased knowledge, skills, attitudes, self-efficacy, interest in future work with the underserved, and interest in primary care among Spanish-speaking PA students after their volunteer work at a student-run free clinic in the United States. As the PA career is a vital part of the US health care system, PA students should continue to be studied in greater depth. Future studies should be longitudinal in nature and explore the study outcomes and career choices of PA students.

## Figures and Tables

**Table 1.** Sociodemographic characteristics of participants (N=56)

Characteristic	Frequency (%)
Female	32 (57.1)
Male	22 (39.3)
Gender: other	2 (3.6)
Age <30 yr	19 (33.9)
Age ≥30 yr	37 (66.1)
Clinical experience of 5 years or less	22 (39.3)
Clinical experience of more than 5 years	34 (60.7)
Speak Spanish	25 (44.6)
Cannot speak Spanish	31 (55.4)
Volunteered 3+times at the free clinic	32 (57.1)
Volunteered less than 3 times at the free clinic	24 (42.9)
First-year student	18 (32.1)
Second-year student	38 (67.9)
Volunteered at a free clinic before starting PA school	32 (57.1)
Non-volunteering	24 (42.9)
Race/ethnicity	
Non-Hispanic white	33 (58.9)
Hispanics	10 (17.9)
Asian	7 (12.5)
Other race	6 (10.7)
Primary work experience before PA school (multiple answers, listed top 3)	
Technician	21 (37.5)
Certified nurse assistant/medical assistant	14 (25.0)
Phlebotomist	8 (14.3)

PA, physician assistant.

**Table 2.** Descriptive statistics of the variables used in regression analysis for each sub-scale

Variable	Mean±standard deviation
Attitudes^a)^	
Knowledge	5.61±1.12
Skills	5.53±1.15
Self-efficacy	5.75±1.03
Effect^a)^	6.10±1.13
Readiness^b)^	4.53±0.59

a) Range, 1–7; higher scores indicate higher levels of knowledge/skills/self-efficacy/effect.

b) Range, 1–5; higher scores indicate higher levels of readiness.

**Table 3.** P-values and confidence intervals for each demographic characteristic according to the response categories of the measurement tool

Variable	Knowledge	Skills/attitudes	Self-efficacy	Interest in future work	Interest in primary care
Female	0.298 (0.304 to 0.970)	0.826 (-0.613 to 0.764)	0.155 (-0.152 to 0.928)	0.301 (-0.160 to 0.508)	0.917 (-0.649 to 0.585)
Age under 30	0.715 (-0.564 to 0.816)	0.262 (-0.325 to 1.166)	0.218 (-0.222 to 0.948)	0.402 (-0.210 to 0.515)	0.063 (-0.036 to1.302)
Clinical experience 5+ years	0.538 (-0.896 to 0.474)	0.946 (-0.765 to 0.715)	0.126 (-1.029 to 0.131)	0.872 (-0.388 to 0.330)	0.765 (-0.762 to 0.564)
Spanish speaker	0.677 (-0.500 to 0.763)	0.552 (-0.479 to 0.885)	0.001 (0.419 to 1.488)	0.052 (-0.003 to 0.660)	0.012 (0.182 to 1.405)
First-year student	0.1 (-0.124 to 1.366)	0.602 (-0.595 to 1.015)	0.096 (-1.164 to 0.098)	0.289 (-0.183 to 0.600)	0.529 (-0.494 to 0.949)
Volunteered at free clinic 3+ times	0.199 (-0.237 to 1.111)	0.798 (-0.635 to 0.821)	0.505 (-0.762 to 0.380)	0.910 (-0.374 to 0.334)	0.821 (-0.727 to 0.579)
White	0.907 (-0.686 to 0.610)	0.420 (-0.983 to 0.417)	0.311 (-0.829 to 0.269)	0.952 (-0.330 to 0.350)	0.820 (-0.557 to 0.699)

## Supplementary materials

Supplement 1. Data files are available from https://doi.org/10.7910/DVN/XR20NQ

Supplement 2. Audio recording of the abstract.
