# Supplementary material for: Higher levels of self-efficacy and readiness for a future career among Spanish-speaking physician assistant students after their volunteer work at a student-run free clinic in the United States
Source: J Educ Eval Health Prof. 2019 Sep 6;16:27. doi: 10.3352/jeehp.2019.16.27 (PMC6805251; doi:10.3352/jeehp.2019.16.27)
Supplement: Supplementary file 2 [file jeehp-16-27-app.pdf]

Appendix 1. Physician assistant student survey

Please rate the effect of volunteering at the Maliheh Free Clinic on you, i.e., Volunteering at Maliheh affected my feeling of caring for underserved populations **a great deal, not at all**, or neutral (did not change my feeling).

	Not at all			Neutral			A great deal
1. My degree of knowledge about the problems of the underserved	1	2	3	4	5	6	7
2. My degree of knowledge about the problems of underserved minority families	1	2	3	4	5	6	7
3. My clinical skills in the care of the underserved	1	2	3	4	5	6	7
4. My clinical skills regarding the problems of underserved minority families	1	2	3	4	5	6	7
5. I feel capable of caring for the underserved	1	2	3	4	5	6	7
6. I feel capable of caring for underserved minority families	1	2	3	4	5	6	7
7. I feel comfortable caring for the underserved	1	2	3	4	5	6	7
8. I feel comfortable caring for underserved minority families	1	2	3	4	5	6	7
9. I believe I can make a difference in the care of the underserved	1	2	3	4	5	6	7
10. My interest in working with the underserved after I graduate	1	2	3	4	5	6	7
11. My interest in being a primary care physician assistant	1	2	3	4	5	6	7

Please rate the effect of volunteering at the Maliheh Free Clinic on your attitudes.

	Very negative			Neutral			Very positive
1. My attitude towards the care of the underserved	1	2	3	4	5	6	7
2. My attitude towards the care of underserved minority families	1	2	3	4	5	6	7

How much do you agree or disagree with the statements below? All of these statements are in regards to **volunteering at the Maliheh Free Clinic.**

	Strongly disagree	Disagree	Neutral	Agree	Strongly agree
1. Learning with other students helps me become a more effective member of a health care team	1	2	3	4	5
2. Patients would ultimately benefit if physician assistant students worked together to solve patient problems	1	2	3	4	5
3. Shared learning with other students will increase my ability to understand clinical problems	1	2	3	4	5
4. Communication skills should be learned with other students	1	2	3	4	5
5. Shared learning will help me to think positively about other professionals	1	2	3	4	5
6. Team-working skills are essential for all health care students to learn	1	2	3	4	5
7. Shared learning will help me to understand my own limitations	1	2	3	4	5

Do you speak Spanish?

☐ Yes, fluent ☐ Yes, a little ☐ No

Do you speak a foreign language (to be able to communicate with patients) other than Spanish?

☐ Yes ☐ No

Are you a first or second year physician assistant (PA) student?

☐ First ☐ Second

How many times did you volunteer at Maliheh?

☐ 1–2 ☐ 3–6 ☐ 7–14 ☐ 15–20 ☐ 21+

Did you volunteer in a free clinic before starting PA school?

☐ Yes ☐ No

How old are you?

☐ 21–29 ☐ 30–39 ☐ 40–49 ☐ 50+

What is your gender?

☐ Female
☐ Male
☐ Non-binary/third gender
☐ Prefer to self-describe ____________________
☐ Prefer not to say

Which do you consider yourself? (Please pick all that apply.)

☐ Asian/Pacific Islander
☐ White–non Hispanic
☐ Hispanic or Latino/Latina
☐ African or African American
☐ American Indian/Alaska Native
☐ Other, please specify: __________________________

What was your primary work experience before PA school? (Please check all that apply.)

☐ Technician (magnetic resonance imaging technician, emergency medical technician, electrocardiogram technician etc.)
☐ Certified nurse assistant/medical assistant
☐ Phlebotomist
☐ Nurse
☐ Foreign medical graduate/doctor
☐ Interpreter
☐ Laboratory work
☐ Public health work
☐ Scribe
☐ Pharmacist
☐ Other; please specify: ________________________

Which of the following described your level of clinical experiences?

☐ 1–3 years ☐ 4–5 years ☐ 6–8 years ☐ 9–15 years ☐ 16 plus years
